# Other PHAC publications

**DOI:** 10.24095/hpcdp.46.2.07

**Published:** 2026-02

**Researchers from the Public Health Agency of Canada also contribute to work published in other journals and books. Look for the following articles published in 2025:**

**Chen SX**, Lee MJ, McVea DA, **Henderson SB**. Antipsychotics and other risk factors for mortality among people with schizophrenia during an extreme heat event: a population-based case-control study. Sci Rep. 2025;15(1):34505. https://doi.org/10.1038/s41598-025-17591-0

John S, Joseph KS, Fahey J, **Liu S**, Lisonkova S, Kramer MS. Do birthweight-for-gestational age centiles predict serious neonatal morbidity and neonatal mortality? Paediatr Perinat Epidemiol. 2025. https://doi.org/10.1111/ppe.70065

Nussbaumer-Streit B, Booth A, **Garritty C**, Hamel C, Munn Z, Tricco AC, et al. Overview of evidence synthesis types and modes. J Clin Epidemiol. 2025;187:111970. https://doi.org/10.1016/j.jclinepi.2025.111970

Walker KL, Gaudreault A, **Prince SA**, Goldfield G, Taler V, Taljaard M, et al. Cycling and cognition in middle-aged and older adults: a scoping review. J Cycl Micromobil Res. 2025;6:100092. https://doi.org/10.1016/j.jcmr.2025.100092

